# Deep Survey of GABAergic Interneurons: Emerging Insights From Gene-Isoform Transcriptomics

**DOI:** 10.3389/fnmol.2019.00115

**Published:** 2019-05-07

**Authors:** Lin Que, Jochen Winterer, Csaba Földy

**Affiliations:** Laboratory of Neural Connectivity, Faculties of Medicine and Natural Sciences, Brain Research Institute, University of Zurich, Zurich, Switzerland

**Keywords:** GABAergic interneuron, single-cell RNA seq, cell type classification, trans-synaptic signaling, gene isoforms

## Abstract

GABAergic interneuron diversity is a key feature in the brain that helps to create different brain activity patterns and behavioral states. Cell type classification schemes—based on anatomical, physiological and molecular features—have provided us with a detailed understanding of the distinct types that constitute this diversity and their contribution to brain function. Over recent years, the utility of single-cell RNAseq has majorly complemented this existing framework, vastly expanding our knowledge base, particularly regarding molecular features. Single-cell gene-expression profiles of tens of thousands of GABAergic cells from many different types are now available. The analysis of these data has shed new lights onto previous classification principles and illuminates a path towards a deeper understanding of molecular hallmarks behind interneuron diversity. A large part of such molecular features is synapse-related. These include ion channels and receptors, as well as key synaptic organizers and trans-synaptic signaling molecules. Increasing evidence suggests that transcriptional and post-transcriptional modifications further diversify these molecules and generate cell type-specific features. Thus, unraveling the cell type-specific nature of gene-isoform expression will be a key in cell type classification. This review article discusses progress in the transcriptomic survey of interneurons and insights that have begun to manifest from isoform-level analyses.

## Introduction

The activity of excitatory neurons is shaped by a highly diverse population of GABAergic interneurons (Klausberger and Somogyi, 2008; Rudy et al., 2011; Kepecs and Fishell, 2014). A clear comprehension of these GABAergic cells in the brain is therefore crucial for understanding the brain circuits in which they participate. To address the complexity of this diverse population, grouping of neurons into distinct cell types is a key to delineate and ultimately understand their function.

Classification of interneurons depends on an unambiguous identification of features that are unique for one cell type compared to others. These features typically encompass morphological and physiological characteristics, as well as molecular markers (Freund and Buzsáki, 1996; Klausberger and Somogyi, 2008; Ascoli et al., 2008; Booker and Vida, 2018). In addition, developmental origin has emerged as another distinctive feature for describing GABAergic cells (Pelkey et al., 2017; Wamsley and Fishell, 2017; Lim et al., 2018). However, whether unique features exist for every potential distinct cellular identity or if, additionally, continuous modes of variables are needed to cover the diversity of interneurons, is an ongoing matter of debate.

Ever since next generation sequencing methods have touched upon the molecular features of cell type classification, an explosion of new information has set the field in motion and thereby increased the expectation to track down the problem of neuronal cell identity (Poulin et al., 2016). The immense power of transcriptomic surveys has not only been highlighted by their capacity to closely match previous cell type classifications, but also in facilitating the discovery of potential new cell types. However, these surveys disclose additional layers of complexity. First, the analysis of large-scale transcriptomic data required introducing the concept of “transcriptomic cell types,” which are defined as clusters of cells with differential gene expression patterns. Differences between clusters may be discrete or continuous (Tasic et al., 2016, 2018; Harris et al., 2018; Muñoz-Manchado et al., 2018). Continuous differences, also referred to as continuous variability, between clusters could be the result of similarities in the molecular heterogeneity of cells from neighboring and even overlapping transcriptomic cell types. Second, transcriptomic surveys revealed that cell states, which may be defined as the cells’ progress through their developmental trajectory (La Manno et al., 2018; Mayer et al., 2018; Mi et al., 2018) or as a consequence of neuronal activity (Tasic et al., 2018), need to be accounted for when classifying single cells. As a result, there is a high level of granularity in identifying single cells, which makes their classification still challenging.

In addition to their ability to detect differential gene expression levels, transcriptomic surveys began to reveal insights into cell type-specific transcriptional and post-transcriptional modifications, such as differential promoter usage and alternative splicing. Consequently, it has been shown that different gene-isoforms can correlate with cell type identity (Fuccillo et al., 2015; Tasic et al., 2016; Karlsson and Linnarsson, 2017; Nguyen et al., 2016; Wamsley et al., 2018).

In this review article, we discuss insights gained from single-cell transcriptomics and how they augment the existing classification schemes of GABAergic cell types. More specifically, we will elaborate on the importance of introducing the analysis of gene-isoform expression levels in cell type taxonomies and how this could facilitate the classification of cell type identities.

## Transcriptomic Cell Types and GABAergic Interneuron Diversity

On-going progress in single-cell transcriptomics provides a major technological driving force for understanding cell-type identity. One of the first single-cell transcriptomic census of neural cell types was done in somatosensory cortex and hippocampus (Zeisel et al., 2015). Since then, molecular profiles of distinct cell types have been characterized in the developing brain to understand the developmental trajectory that gives rise to the high diversity of cell types (Mayer et al., 2018; Rosenberg et al., 2018; Zhong et al., 2018). In the adolescent and adult brain, molecular profiling studies in the hippocampus, dorsal striatum and different cortical areas have started to establish an indispensable framework of static and dynamic transcriptomic states behind cell taxonomies (Tasic et al., 2016, 2018; Harris et al., 2018; Muñoz-Manchado et al., 2018).

The hippocampus is one of the most investigated circuitry in the brain and therefore provides a well-established reference of morphological, immunohistochemical and electrophysiological characterizations. Transcriptional profiling of single-cell samples from hippocampal CA1 interneurons using the pan-GABAergic Slc32a1-Cre line revealed 10 major GABAergic “transcriptomic continents” (Harris et al., 2018), which further differentiated in 49 transcriptomic interneuron types. Referencing single-cell gene expression patterns to the extensive knowledge base of the hippocampus revealed that the 49 transcriptomic clusters were organized according to the previously described 23 interneuron classes. However, due to an overlap of gene expression levels of cells that initially fall into different clusters, transitions between populations remained and a continuous variability persisted throughout the dataset.

In another categorizing study in the dorsal striatum, transcriptomic analysis of GABAergic cells could not rely on back referencing to an existing knowledge-base because an elaborated description of resident interneurons was not available (Muñoz-Manchado et al., 2018). Using *Htr3a, Lhx6* and *Pvalb* specific transgenic lines, this study revealed discrete cell types, albeit fewer than within the hippocampus. In contrast to what was found in the hippocampus (Harris et al., 2018), *Pvalb* expressing interneurons did not cluster into one or more discrete groups, but continuously rendered into a larger population of *Pthlh* expressing cells that displayed a spatial gradient of *Pvalb* expression levels along the dorsoventral and mediolateral axis of the dorsal striatum. In addition, this gradual *Pvalb* expression was likewise reflected in electrophysiological characteristics of these cells, where higher expression levels of *Pvalb* correlated with fast-spiking signatures, such as shorter action potential half-width.

To study cell classification in the primary visual cortex, Tasic et al. (2016) chose for more detailed, pre-determined specificity by using 25 different *Cre*-driver lines. This allowed for a selection of specific subsets of cortical cells and access to both abundant as well as rare cell types. Differential gene expression analysis revealed robust separation of cells into 49 clusters, incidentally the same number as in hippocampus study, of presumed GABAergic, glutamatergic and non-neuronal cell types. These clusters were based on both cells that were consistently classified into the same cluster and cells of which their identity could not be determined using clustering algorithms, respectively referred to as core and intermediate cells. Discreteness of clusters largely depended on core cells, whilst intermediate cells caused a continuous gene expression variation. Nonetheless, identities of both core and intermediate cells could be inferred from referencing to previous studies and knowledge about the cre-driver line from which the cell was collected. Subsequent to identifying transcriptomic cell types, examination of transcriptional and post-transcriptional modifications revealed differential exon usage for 567 exons in 320 genes in a cell type-specific manner, indicating that isoform-level analyses could further facilitate the classification of cell types (see also Karlsson and Linnarsson, 2017).

In a more recent article, Tasic et al. (2018) revisited the primary visual cortex, and in addition studied the anterior lateral motor cortex in mice. An extensive dataset of 23,822 single-cell transcriptomes was created, based on 47 cre-driver lines. This allowed definition of 133 transcriptomic neuronal cell types (61 GABAergic) in both areas. Similar to their previous article, both discrete and continuous gene expression in the dataset was observed. Interestingly, while the heterogeneity in layer 4 cells was previously accounted for by both core and intermediate types (Tasic et al., 2016), this new survey rendered them as one continuous type without separable features, possibly owing to higher cell numbers and improved gene detection.

In contrast to large-scale approaches, patch pipette-based transcriptomics has been introduced as a method to combine electrophysiological with transcriptomic analyses. First, probe-based single-cell RT-PCR, qRT-PCR and whole-genome microarrays were performed after manual cell picking with a patch-pipette, either with or without electrophysiological recordings (Geiger et al., 1995; Okaty et al., 2009; Tricoire et al., 2011; Fuccillo et al., 2015). More recently, single-cell RNAseq approaches were introduced, allowing an unbiased access to all RNA species (Cadwell et al., 2016; Fuzik et al., 2016; Földy et al., 2016). Although manual cell picking results in a lower throughput, it grants *ad hoc* electrophysiological and morphological confirmation of the cell. Consequently, patch pipette-based transcriptomics enables the correlation of electrophysiological properties (e.g., spike patterns) to the molecular profile (e.g., ion channel composition) of the cell of interest, as has already been shown in several studies (Fuzik et al., 2016; Földy et al., 2016; Muñoz-Manchado et al., 2018). Furthermore, it enables a transcriptomic analysis of connected neurons, as evidenced by electrophysiological signatures of synaptic transmission, and molecules that define circuit connectivity motifs (Földy et al., 2016). In addition, recent studies introduced the use of driver lines that more precisely label specific morphological cell types, which grants a distinction that allows direct comparisons between these types (Paul et al., 2017; Favuzzi et al., 2019). These studies revealed cell type-specific expression of synaptic molecules, highlighting their close relation to cell type identity and connectivity (Földy et al., 2016; Paul et al., 2017; Favuzzi et al., 2019). Because these methods do not require clustering-based inferences and rely less on back referencing to an existing knowledge base, such approaches offer a straightforward access to the transcriptomic signature of cell types.

## Challenges Associated With Interpreting Transcriptomic Cell Types

There are several challenges that need to be addressed when interpreting single-cell RNAseq data. First, RNAseq captures only a static snapshot at the time when the cell is collected. With the aim to address this snapshot nature of single-cell transcriptomes, based on the fraction of spliced vs. un-spliced RNA, RNA velocity analysis can predict the future state of developing cells at a timescale of hours, and describe fate decisions of major neural lineages in the hippocampus (La Manno et al., 2018). Furthermore, distinct cell states may influence transcriptomic profiles by forming “temporal” clusters. For example, the comparison of single-cell transcriptomes collected from dark-reared vs. light-exposed animals revealed that several glutamatergic and GABAergic types displayed statistically significant enrichment or depletion of early- and/or late-response genes (Tasic et al., 2018).

Second, the detected discreteness or continuity between clusters largely depends on technical parameters, such as gene detection, cell sampling, and the stringency of clustering criteria (Harris et al., 2018; Tasic et al., 2018). In addition, different clustering algorithms revealed different grouping of cells, as is highlighted by the comparison between algorithms of two studies (Tasic et al., 2016; Harris et al., 2018). Therefore, the identification of intrinsic biological variations remains challenging. Considering the pace at which transcriptomic methods continue to develop, and the number of sequenced cells continues to increase, it is likely that these problems will have to be revisited upfront in the light of new data.

**Figure 1:**
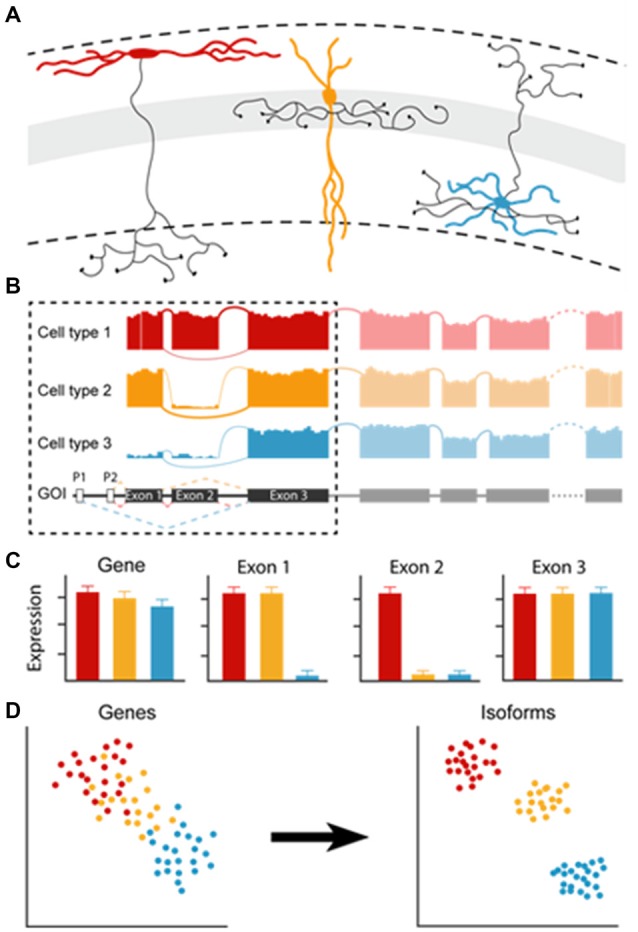
The effect of cell type-specific isoform expression on gene expression pattern analysis. **(A)** Examples of different cell types based on their morphological appearance. **(B)** Modifications such as alternative splicing (cell type 2) or differential promotor usage (cell type 3) generate different isoforms. **(C)** Large differences in isoform length may result in apparent differential gene expression, small differences go unnoticed (“Gene”). However, looking at cell type-specific differential exon usage would reveal distinctions between cell types (in “Exon1” and “Exon2”). **(D)** Clustering by gene expression may result in cell type clusters with continuous variability (“Genes”). Clustering of the same cells based on isoform usage could possibly result in discrete clustering (“Isoforms”).

Current classification efforts using single-cell transcriptomic data rely on neuronal clusters that are generated based on differential gene-expression but not on gene-isoform analysis. However, even in single genes, cell-intrinsic gene editing during transcription, the use of different promoters and alternative splicing sites can generate a level of diversity that affects gene-expression level readouts, and thereby cell classification. When cell types differentially express isoforms of a single gene of interest, all cells will be recognized as expressing that gene and will not be separated into different clusters. However, using single-cell isoform RNA-seq, a recent study identified more than 10,000 RNA isoforms in cerebellar cell types, and cell type-specific combination patterns of distant splice sites, indicating that many isoforms exist in a cell type-specific fashion (Gupta et al., 2018). By looking at differential gene expression, isoforms would reveal at most apparent gene expression differences, when differences in length between isoforms are large. Cell type-specific RNA modifications may be read out as a minor difference in the gene’s expression while exerting a major impact in determining or correlating extremely well with cell identities. Depending on the method that is implemented, processing of single-cell samples can result in partial or full-length recovery of RNA molecules (Stegle et al., 2015). Although partial recovery is sufficient for the detection of genes, application of full length-cDNA would allow going beyond gene-expression analyses and examine the expression of different gene isoforms in detail. Partial and full-length recovery of RNA can be applied in both large-scale as well as pipette-based approaches (demonstrated in Fuccillo et al., 2015; Földy et al., 2016; Tasic et al., 2018). If all isoforms would be included in clustering analyses, would cell types clearly subdivide (Figure 1)?

## Isoforms That Make the Difference

**Figure 2:**
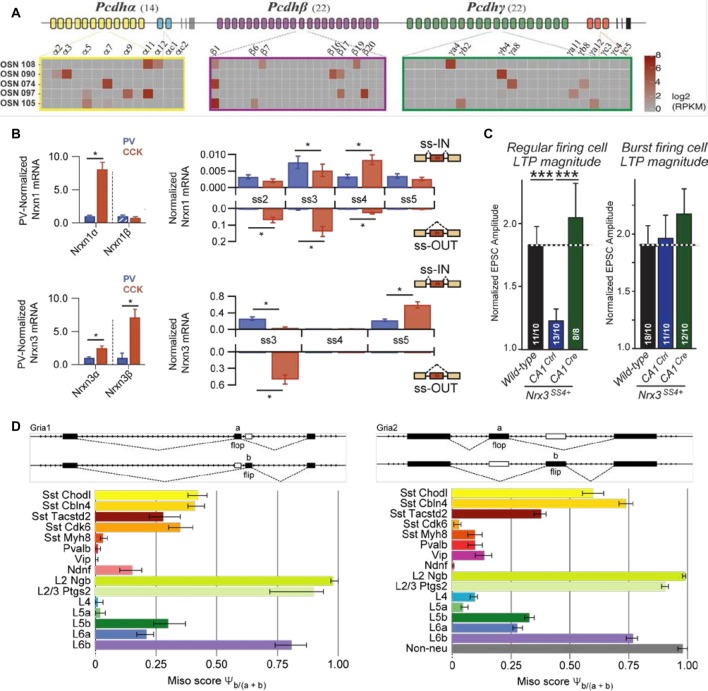
Examples of cell- and cell type-specific isoform expression.** (A)** Protocadherin isoforms may be divided in two categories, the alternate (yellow, green and purple), and the C type (blue and red). Shown here are single-cell expression examples of the protocadherin alpha/beta/gamma in five cells, where the presence of individual protocadherin isoform mRNA is represented by a gradient of gray (no expression) to red (high expression; adapted from Mountoufaris et al., 2017 with permission). **(B)** Left: neurexin alpha/beta isoform expression in hippocampal *Cck (CCK)* and *Pvalb (PV)* GABAergic cells, normalized to the average level in *Pvalb (PV)* cells. Right: splice-site graph that displays averaged single-cell splice isoform expression values for either being “spliced in” (up-ward bars) or “spliced out” (down; adapted from Fuccillo et al., 2015 with permission). **(C)** Averaged EPSC amplitudes, recorded from subiculum neurons and elicited by electrical stimulation of CA1 pyramidal cells, over the last 10 min of LTP recordings and normalized to the baseline for cells of inactive (“Ctrl”) or active excision of SS4 (“Cre”). The magnitude of LTP (“Ctrl”) depended on the identity of postsynaptic cells; left graph represents postsynaptic regular firing cells, right graph postsynaptic burst firing cells (adapted from Aoto et al., 2013 with permission). **(D)** Both *Gria1* and *Gria2* display similar, but cell type-specific alternative exon usage of “flip” and “flop,” which suggests a shared mechanism for alternative splicing (adapted from Tasic et al., 2016 with permission). ***P < 0.001, *indicates significant difference between groups (*P* < 0.05; Mann-Whitney U test).

Transcriptional and post-transcriptional RNA modifications greatly extend on the molecular repertoire (Barash et al., 2010; Gabut et al., 2011; Zhang et al., 2016; Mauger and Scheiffele, 2017; Gandal et al., 2018; Wamsley et al., 2018) that may also generate cell type-specific features. For example, clustered protocadherins, which are important for determining cell identity and circuit assembly of olfactory sensory neurons, display cell-specific promoter usage (Figure 2A; Lefebvre et al., 2012; Chen W. V. et al., 2017; Mountoufaris et al., 2017). In addition, isoforms of cell adhesion molecule neurexins have been demonstrated to show cell type specificity between hippocampal neurons (Figure 2B; Fuccillo et al., 2015; Nguyen et al., 2016), and between nucleus accumbens projection and nucleus accumbens targeting neurons (Fuccillo et al., 2015). Furthermore, as is the case for alternative splicing of neurexin-3 in CA1 neurons, inclusion/exclusion of a single-cassette exon renders a lack or presence of long-term plasticity in output synapses depending on the identity of postsynaptic subicular cell type (Figure 2C; Aoto et al., 2013). Finally, due to gene editing, two consecutive exons (“flip” and “flop”) in *Gria1* and *Gria2* displayed cell type specificity in primary visual cortex between layer 2/3 (flip) vs. layer 4 pyramidal cell types (flop; Figure 2D; Tasic et al., 2016). This difference renders a high Ca^2+^ permeability and mediates significant Ca^2+^ influx (flip) or lack thereof (flop; Sommer et al., 1990; Lomeli et al., 1994; Geiger et al., 1995). These examples illuminate that the lack of inclusion of different isoforms, which could have the capacity to disambiguate cell types, potentially underpowers current transcriptomic surveys.

Below, we describe emerging evidence on GABAergic signaling, which suggests that a large isoform diversity exists both pre- and post-synaptically. As synaptic molecules exist at the intersection of connectivity and cell identity, isoform diversity could possibly reflect distinct cell types. If so, isoform specificity could serve as a platform for cell type classification.

## Gene Isoforms in GABAergic Signaling

Within the synapse, cell surface molecules mediate transsynaptic signaling and are emerging as key organizers of synapse function (de Wit and Ghosh, 2016; Südhof, 2017). There is an increasing interest in understanding how key synaptic organizers drive circuit assembly for GABAergic interneurons and correlate with cell type identity (Földy et al., 2016; Li H. et al., 2017; Paul et al., 2017; Favuzzi et al., 2019). Such molecules may ubiquitously dictate the establishment and maturation of GABAergic synapses (e.g., neuroligin-2 and Slitrk-3; Li J. et al., 2017), or have been proposed to display cell type-specific expression.

Presynaptically, neurexins are key organizers that are expressed in thousands of alternatively spliced isoforms (Schreiner et al., 2014, 2015; Treutlein et al., 2014) and have been shown to restrict and rank trans-synaptic binding preference to their postsynaptic partners (Takahashi and Craig, 2013; de Wit and Ghosh, 2016; Südhof, 2017). Transcriptomic analysis of a few isoforms and cell types has begun to reveal that cell type-specific expression of neurexins may be important for brain function (Fuccillo et al., 2015; Traunmüller et al., 2016; Chen L. Y. et al., 2017; Nguyen et al., 2016). Intriguing differences in alternative splicing have been shown for hippocampal *Pvalb* and *Cck* interneurons. Especially, the single exon cassette at alternative splicing site 3 was uniformly retained in *Pvalb* interneurons and spliced out in *Cck* interneurons. Although the impact of neurexins on the build-up of the presynaptic active zone remains elusive, this striking difference in synapse organizing molecules is reflected in the diametrically different use of presynaptic Ca^2+^-channels and release-modulating receptors by these interneurons (Freund and Katona, 2007). In addition, neurexin alternative splicing might also affect transsynaptic binding and thereby synapse function: while loss of neurexin-ligand neuroligin-3 function affects inhibitory postsynaptic potentials from *Cck* interneurons by disinhibition of tonic endocannabinoid signaling, synaptic transmission from *Pvalb* interneurons remains unaltered (Földy et al., 2013). In the prefrontal cortex, genetic deletion of all neurexins in *Pvalb* interneurons decreased synapse numbers without affecting GABA release in surviving synapses while in *Sst* interneurons, the same manipulation impaired presynaptic Ca^2+^ influx without changing synapse numbers (Chen L. Y. et al., 2017). However, neurexin isoform expression has not been identified in these cells.

Postsynaptically, gephyrin and collybistin are key organizers for the GABAergic density. Their roles were first highlighted by experiments in which forced expression of synaptogenic cell-adhesion molecule neuroligin-2 lead to gephyrin and collybistin aggregation and recruitment of GABA receptors (Poulopoulos et al., 2009). Gephyrin is subject to extensive alternatively splicing in at least 10 canonical sites (Prior et al., 1992; Paarmann et al., 2006), which can potentially generate a large number of possible isoforms. Alternative splicing of gephyrin occurs predominantly in the C-domain, where most phosphorylation sites have been reported. Because phosphorylation ultimately determines gephyrin folding and clustering (Zacchi et al., 2014), it is conceivable that different isoforms generate different gephyrin function. For collybistin, three major C-terminal isoforms have been described (CB1-3, Harvey et al., 2004). While distinct CB2 isoforms regulate translocation of gephyrin to the cell surface and formation and maintenance of gephyrin clusters at GABAergic sites (Tyagarajan et al., 2011), the CB1 isoform has been shown to selectively facilitate gephyrin clustering at distal portions of immature dendrites (de Groot et al., 2017). Alternative splicing in both the CB1 and CB2 isoforms have been observed (Harvey et al., 2004; de Groot et al., 2017). However, cell type-specific expression of these gephyrin/collybistin isoforms has not been investigated.

Parallel to alternative splicing, expression of neurexin splicing regulators *SLM1* *and SLM2* appear in a cell type-specific manner (Iijima et al., 2014). Similarly, reports on gephyrin splicing factors *Nova1* and *Nova2* also indicate cell type specificity (Yuan et al., 2018). Intriguingly, alternative splicing of neurexins, gephyrin and collybistin are commonly regulated by *Sam68*, which, in contrast to the above factors displays ubiquitous expression (Witte et al., 2018).

Isoform diversity of synaptic molecules plays an important role in synapse connectivity. These synaptic connections are at the base of neuronal connectivity, which in turn defines the anatomical identity of a cell. Therefore, the transcriptomic identity, including cell type-specific isoform diversity, may correspond well with the anatomical identity of a cell (Li H. et al., 2017). To this end, single-cell transcriptomics would allow a precise, cell type-specific isoform dissection of synaptic molecules associated with GABAergic synapses, and give insight into the transcriptomic and anatomical identities of distinct cell types.

## Neuroanatomy-Based Interpretation of Transcriptomic Cell Types

Cajal’s work has become the foundation for the classification of neurons based on morphological characteristics and although behavior-specific activity of cell types has become an almost unambiguous marker for identifying GABAergic cell types (Klausberger and Somogyi, 2008; Kepecs and Fishell, 2014), specific axonal and dendritic features still provide fundamental reference points in cell type classification. As highlighted above, transcriptomic surveys are closing the gap between genetic content and cell identities. In this effort, inferences made between transcriptomic cell types and an existing knowledge base have played crucial roles. In specific cases, however, it appears to be inevitable to generate transcriptomic data directly from anatomically-defined cells. Development of transgenic lines that specifically label anatomically-defined cell types will be important for direct comparisons between the transcriptomic signatures of these cells. Patch pipette-based transcriptomics could largely facilitate the development of such lines, as it potentially allows identification of driver genes behind anatomical features.

Thus far, transcriptomic cell type definitions based on differential gene expression alone have not been consistently able to relate all transcriptomic cell types to specific anatomical features. For example, hippocampal *Pvalb* interneurons, basket and bistratified cells display non-overlapping axonal projections (to pyramidal layer and to oriens/radiatum layers, respectively; Klausberger and Somogyi, 2008), but this fundamental difference could not be resolved as distinct clusters (Harris et al., 2018). In this specific case, the existence of two types of *Pvalb* basket cells adds an extra layer of complexity. In one, soma are located within or in close proximity of the pyramidal layer and their dendrites extend radially along the oriens-radiatum axis (Booker and Vida, 2018). By contrast, soma of the other type are located in the oriens layer and their dendrites run horizontally within the oriens (Maccaferri, 2005), implicating different engagement in CA1 microcircuitry and therefore different function. It is important to note, that the same survey distinctly separated axo-axonic *Pvalb* cells, which establish synapses on the axon initial segment of pyramidal cells. If and how this molecular difference relates to different function remains a question. These transcriptomic insights appear thus far to support an alternative view on the classification of *Pvalb* cells, which suggests that non axo-axonic *Pvalb* neurons comprise a morphologically continuous cell class in which the above defined types represent only prototypes with higher preponderance, but to which not all *Pvalb* cells can be unambiguously assigned based on morphology. Therefore, a combined comprehension of their molecular and morphological identity would be important. Such combined comprehension is equally important for characterizing potential new cell types (Boldog et al., 2018). Overall, combined electrophysiology, anatomical tracing, and molecular profiling that is now equipped with transcriptomics, is defining new standards for cell type discovery.

Finally, a question that still remains is when and how distinct interneuron subtypes, such as the above defined *Pvalb* types, gain their final identity and how this is reflected in their transcriptome. One hypothesis suggests that that interneuron identity is determined at the cell’s birth (“progenitor hypothesis”). Another suggests that progenitors first establish a cardinal identity, that is later refined into a definitive identity through post-mitotic determinants (“progressive maturation hypothesis”; Wamsley and Fishell, 2017). Single-cell RNAseq surveys on anatomically-defined cells over the course of maturation would provide insights into the anatomical as well as the transcriptomic maturation process over time. It would allow the examination of transcriptome dynamics, whether transcriptomic cell types are consistently distinct, or only display a temporal distinction, and how this relates to the anatomical maturation of the cell.

To fully understand the intricacy behind interneuron diversity, single-cell transcriptomics provides a platform to identify the deep molecular architecture determining ontogenesis, morphology and synaptic connectivity of distinct cell types. It has hereby a tremendous influence on our current stand on cell type classification and identity. It not only reflects, but additionally complements previous knowledge on classification based on electrophysiology and morphology, and facilitates cell type discovery. Capitalizing on the capacity of transcriptomic surveys to study transcriptional and post-transcriptional RNA modifications will highlight its potential in understanding cell type-specific molecular mechanisms underlying connectivity, synapse function and cell identity.

## Author Contributions

LQ, JW and CF wrote the manuscript.

## Conflict of Interest Statement

The authors declare that the research was conducted in the absence of any commercial or financial relationships that could be construed as a potential conflict of interest.
